# Witnessing magnetic twist with high-resolution observation from the 1.6-m New Solar Telescope

**DOI:** 10.1038/ncomms8008

**Published:** 2015-04-28

**Authors:** Haimin Wang, Wenda Cao, Chang Liu, Yan Xu, Rui Liu, Zhicheng Zeng, Jongchul Chae, Haisheng Ji

**Affiliations:** 1 Space Weather Research Laboratory, New Jersey Institute of Technology, University Heights, Newark, New Jersey 07102-1982, USA; 2 Big Bear Solar Observatory, New Jersey Institute of Technology, 40386 North Shore Lane, Big Bear City, California 92314-9672, USA; 3 Department of Geophysics and Planetary Sciences, CAS Key Laboratory of Geospace Environment, University of Science and Technology of China, Hefei 230026, China; 4 Collaborative Innovation Center of Astronautical Science and Technology, China; 5 Department of Physics and Astronomy, Astronomy Program, Seoul National University, Seoul 151-747, Korea; 6 Purple Mountain Observatory, Chinese Academy of Sciences, Nanjing 210008, China

## Abstract

Magnetic flux ropes are highly twisted, current-carrying magnetic fields. They are crucial for the instability of plasma involved in solar eruptions, which may lead to adverse space weather effects. Here we present observations of a flaring using the highest resolution chromospheric images from the 1.6-m New Solar Telescope at Big Bear Solar Observatory, supplemented by a magnetic field extrapolation model. A set of loops initially appear to peel off from an overall inverse S-shaped flux bundle, and then develop into a multi-stranded twisted flux rope, producing a two-ribbon flare. We show evidence that the flux rope is embedded in sheared arcades and becomes unstable following the enhancement of its twists. The subsequent motion of the flux rope is confined due to the strong strapping effect of the overlying field. These results provide a first opportunity to witness the detailed structure and evolution of flux ropes in the low solar atmosphere.

## Abstract (web summary)

Understanding the behaviour of magnetic flux ropes in the Sun is crucial for explaining solar phenomena such as flares and space weather. Exploiting the high resolution available in the 1.6 m New Solar Telescope, Wang *et al.* capture the evolution of a flaring twisted flux rope in the low solar corona.

## Graphical abstract

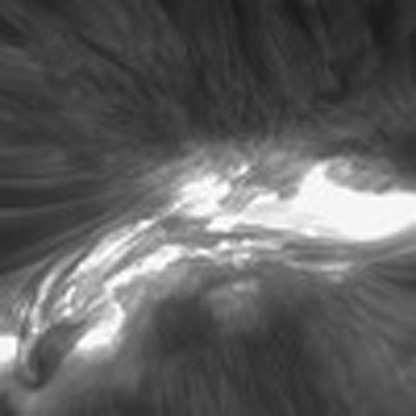

Magnetic flux ropes are a group of twisted magnetic fields writhing about each other and rotating around a common axis. The importance of these helical, current-carrying magnetic flux systems was demonstrated long ago in plasma physics laboratories, such as in the experimental and modelling research with the tokamak^1^,^2^. As the toroidal plasma current in a tokamak produces a hoop force that expands the twisted fields along the major radius, the tokamak and similar experiments were designed to constrain this expansion by adding a strapping field, reducing the effect of the torus instability. Twisted flux ropes are also subject to kink instability^3^, where the rope axis itself can evolve into a helical structure when the flux rope twist reaches a certain threshold^4^. The structure of magnetic flux ropes associated with solar eruptions and interplanetary activities have received significant attention with the application of basic plasma physics. For example, the linkage between the solar plasma and tokamaks was discussed recently^5^. In the large scale, magnetic flux ropes are found in the interplanetary magnetic clouds^6^, which may interact with Earth's magnetic field to generate geomagnetic storms. Near the solar surface, there are evidences that different branches of magnetic loops could reconnect and form a flux rope during the eruption process^7^,^8^,^9^. On the other hand, some studies also suggest that flux ropes are built gradually and may already exist before eruptions^10^,^11^.

Solar magnetic configurations prone to eruptions have been of great importance for space weather, which increasingly affects the modernization of human activities. There are a number of models that describe the initiation mechanism of solar eruptions, such as catastrophe or instability^12^ and magnetic breakout^13^. One of the eruptive magnetic field configuration related to flux ropes is the so-called sigmoid^14^: an S- or inverse S-shaped magnetic field structure as seen in soft X-rays^15^ and extreme ultraviolet^16^. In a qualitative cartoon picture^17^, the onset of a sigmoid eruption begins when the highly sheared inner legs of the two branches of the sigmoidal fields are joined via magnetic reconnection^18^. This initial stage of eruption produces a low-lying shorter loop across the magnetic polarity inversion line (PIL) and a longer twisted flux rope linking the two far ends of the sigmoid. The next stage proceeds when the formed flux rope becomes unstable (due to, for example, the torus instability) and erupts outward to become a coronal mass ejection. However, the insufficient spatial resolution of coronal images predominantly used by previous observational studies of flux ropes seriously hampers an unambiguous and detailed identification of flux ropes and their relation to eruptions. Moreover, the evolution of flux ropes has not been observed in the low solar atmosphere such as the chromosphere.

Here we report on clear observational evidence of a flaring twisted flux rope, taking advantage of H-alpha and He I 10,830 Å images at high resolution (60 and 100 km, respectively) obtained by the recently built 1.6 m New Solar Telescope^19^,^20^ (NST) at Big Bear Solar Observatory (BBSO). NST achieves the high-spatial resolution observation in the chromospheric wavelengths at a high time cadence (∼15 s), thus providing a unique opportunity for a fine-scale assessment of evolving structures in the low atmosphere. An overall inverse S-shaped flux bundle (SFB) is initially observed to lie along the magnetic PIL, from which a set of loops appear to peel off and grow upward into a multi-stranded twisted flux rope within about 2 min. In the meantime, compact brightenings at the footpoints of the loop threads extend to form two elongated flare ribbons. These observations are supplemented by a nonlinear force-free field (NLFFF) extrapolation model^21^ based on the surface vector magnetogram taken by the Helioseismic and Magnetic Imager (HMI) telescope^22^ on board the Solar Dynamic Observatory. Hard X-ray (HXR) observation of the flare recorded by the Reuven Ramaty High Energy Solar Spectroscopic Imager (RHESSI) telescope^23^ is also used to reveal the likely energy release sites. We suggest that in this event, a low-lying single flux rope is embedded in sheared arcades. Following the enhancement of its twists, possibly driven by sub-surface motion, the flux rope becomes unstable. It is also possible that the two groups of the closely lying sheared arcades could reconnect to contribute to the active flux rope. The subsequent motion of the flux rope is, however, confined because of the strong strapping effect of the overlying field. These significant results provide a first opportunity to witness the detailed structure and evolution of flux ropes in the low solar atmosphere.

## Results

### BBSO/NST observation

**Figure 1:**
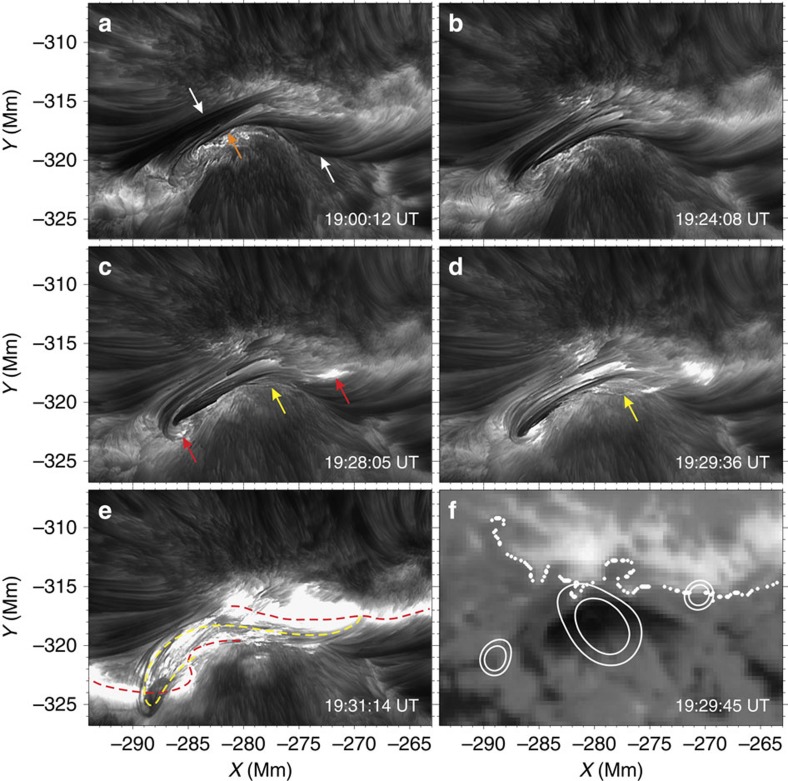
Magnetic flux rope evolution in H-alpha line centre and surface magnetic field structure. The observation is for NOAA Active Region 11817 on 11 August 2013, with image time shown in the bottom right of each panel. (**a**–**e**) BBSO/NST VIS images at the H-alpha line centre. The white (orange) arrows in **a** indicate the weakened (enhanced) arcade loops before the flux rope activity. The red arrows in **c** point to footpoint brightenings. The yellow arrows in **c** and **d** and the yellow dashed line in **e** trace the active flux rope. The red dashed line in **e** delineate the two flare ribbons. (**f**) A corresponding line-of-sight magnetic field observed with Solar Dynamic Observatory/HMI, with the white (black) colour representing positive (negative) field (scaled from −2,000 to 2,000 G) and the dotted line indicating the PIL. The 25–50 keV HXR map is integrated from 19:29:45 to 19:30:45 UT, showing a coronal (the central contours) and two chromospheric footpoint-like (the left and right smaller contours) HXR-emitting sources of the associated flare. The full sequence of BBSO/NST H-alpha centre-line images is provided in Supplementary Movie 1.

**Figure 2:**
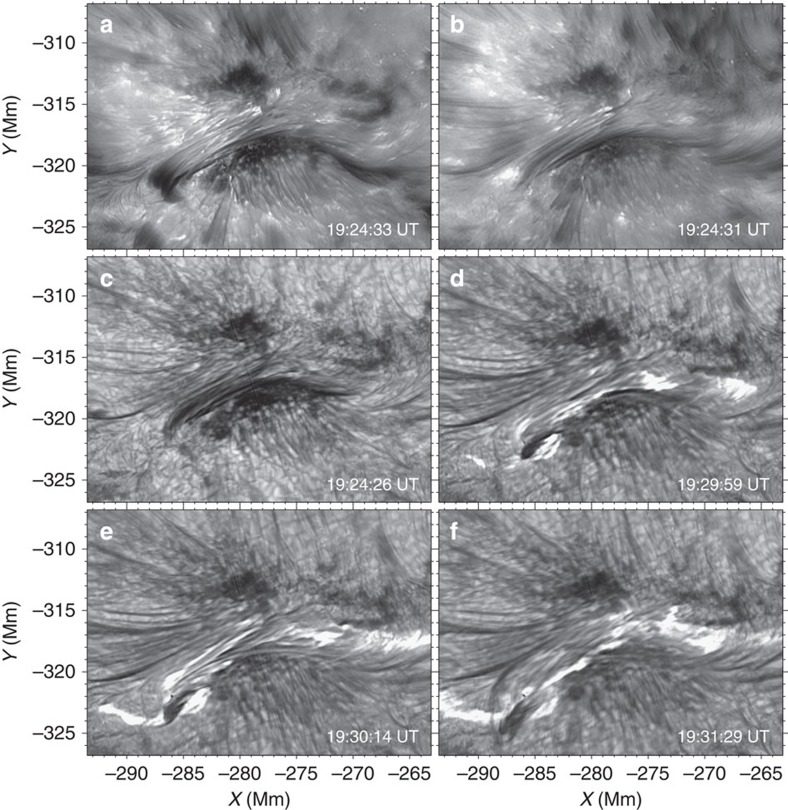
Magnetic flux rope evolution in H-alpha off-bands and He I 10830 Å. BBSO/NST observation on 11 August 2013, including VIS images at the H-alpha red-wing (+0.6 Å) (**a**) and blue-wing (−0.6 Å) (**b**) together with infrared imaging magnetograph (IRIM) images at the He I 10830 Å line (**c**–**f**). These are observed at a lower height than the H-alpha line centre images shown in Fig. 1. It is notable that both H-alpha off-bands (**a**,**b**) and He I 10830 Å (**c**) images right before the event onset exhibit an inverse S-shaped flux rope structure at the centre, bearing a possible similarity to the modelled low-lying red field lines in Fig. 3. The active flux rope on top of the extending flare ribbons are clearly seen in (**d**–**f**). Note that some linear features running at an angle across the centre of the flux rope in **c**–**f** are artifacts produced by the infrared camera. The full sequences of H-alpha off-band images are provided in Supplementary Movies 2 and 3.

On 11 August 2013, we observed an active magnetic flux rope and its associated GOES-class C2.1 flare around 19:30 UT in NOAA Active Region 11817. We present in Fig. 1 the H-alpha line-centre images at selected instances and a corresponding magnetic field on the surface. Owing to the unprecedented high resolution of NST, great details can be visualized. The full dynamics of evolution can be better seen in Supplementary Movie 1. It can be easily recognized that initially there is an overall inverse SFB lying along the PIL (the white dotted line in Fig. 1f). This SFB mainly consists of two branches of sheared arcade loops that seemingly cross and join near the centre (Fig. 1a). From about 19:15 UT (12 min before the event), the outside of these two branches of arcades (especially the eastern one; pointed to by the white arrows) appear to become weakened, while the inner loops (pointed to by the orange arrow) are enhanced (cf. Fig. 1a,b). At the beginning of the event, a bunch of long loop threads start to peel off from the SFB (pointed to by the yellow arrows in Fig. 1c,d). These threads seem to wrap around the entire SFB, with obvious brightenings seen at their two footpoints (pointed to by the red arrows) near the two ends of the SFB. In about 2 min, the threads rapidly expand and rise upward, and grow into a multi-stranded field structure with apparent twist and writhe clearly representing a flux rope (traced by the yellow dashed line in Fig. 1e). As clearly witnessed by BBSO/NST, we can estimate that this flux rope possesses a twist of at least one turn from end to end. In the meantime, the initial compact brightenings in H-alpha have extended in the horizontal direction to form two bright ribbon-like patches (delineated by the red dashed line in Fig. 1e) near the two ends of the rope. A similar evolution of the SFB and the flux rope can be observed with the He I 10,830 Å images (see Fig. 2c–f). Very interestingly, compared with the H-alpha line-centre wavelength, the SFB in He I 10,830 Å and also the H-alpha offbands (±0.6 Å; see Fig. 2a,b and Supplementary Movies 2 and 3) appears more like an integrated single structure right before the event onset.

### Magnetic field structure

**Figure 3:**
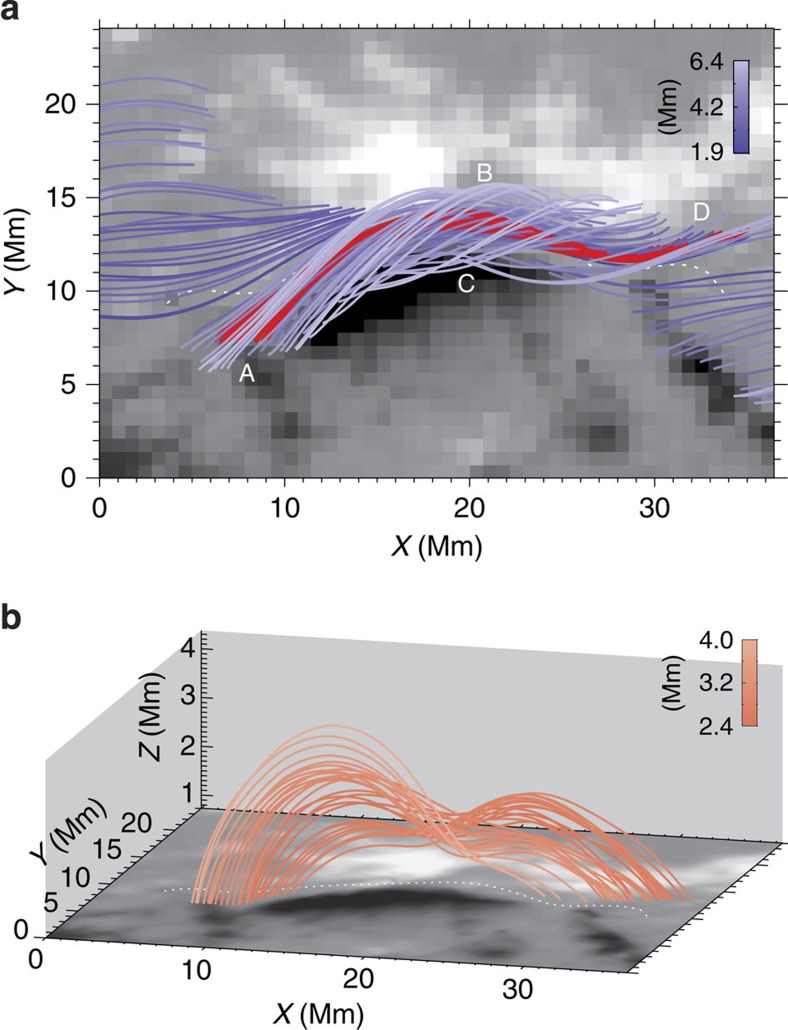
Modelled of magnetic field structure. (**a**) Selected field lines from a NLFFF extrapolation model at 11 August 2013 19:22:24 UT, before the flux rope activity and the associated C2.1 flare. The model is based on the HMI vector magnetic field data (2 × 2 rebinned) in cylindrical equal area coordinate. The blue field lines have a maximum height ≲6.4 Mm and portray the overall inverse SFB. The long red field lines have a maximum height ≲4.0 Mm and run through the SFB. (**b**) A side view of the red field lines. The white dotted line in both the panels is the portion of the PIL above which we compute the average profile of decay index (see Fig. 5).

**Figure 4:**
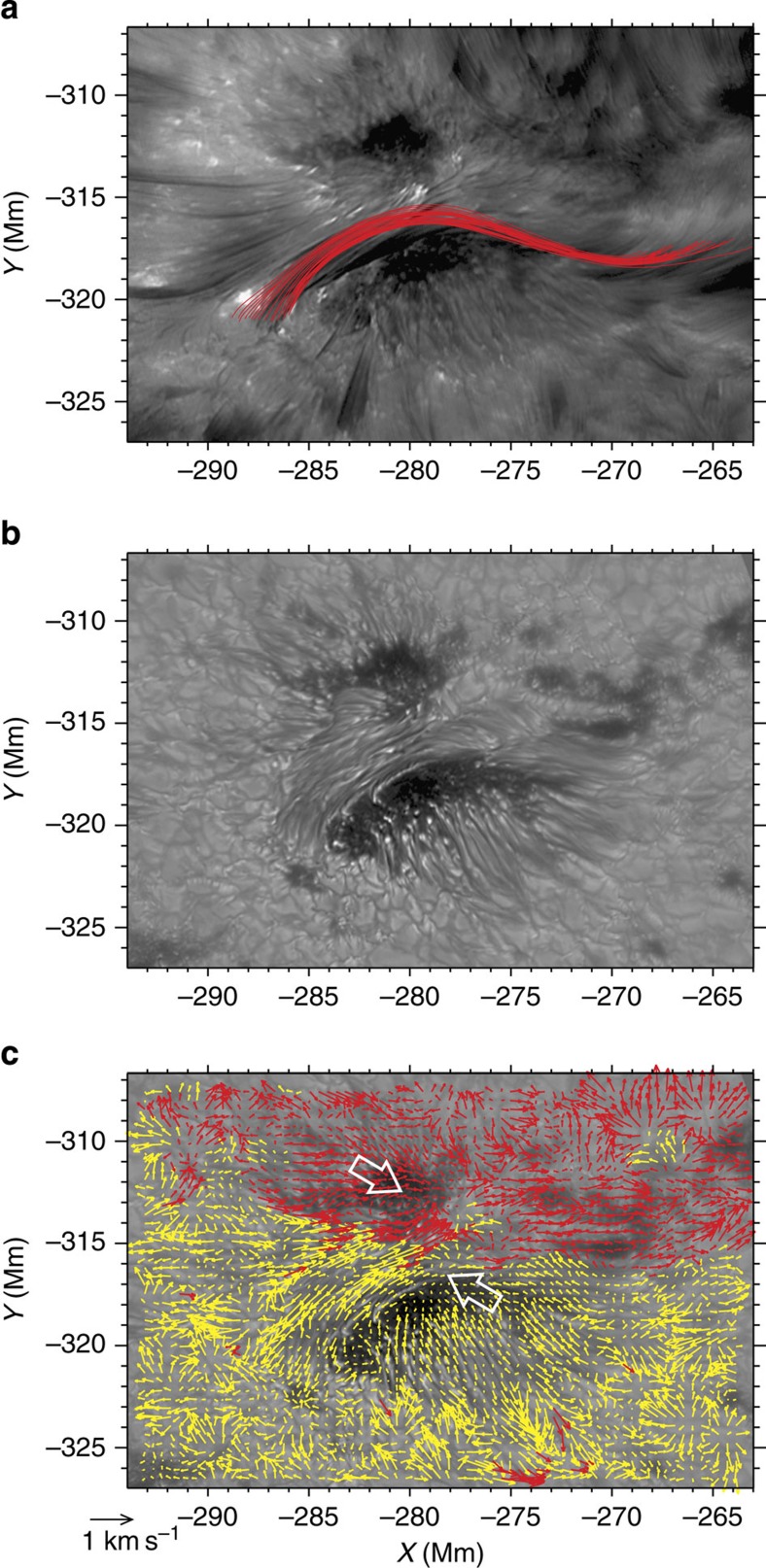
Magnetic flux rope and photospheric flow field. (**a**) The same red field lines as in Fig. 3a but transformed to the image coordinate and overplotted onto a cotemporal pre-flare BBSO/NST H-alpha blue-wing image at 19:22:30 UT, showing a reasonable agreement. (**b**) A BBSO/NST TiO image of the active region at 19:12:04 UT. (**c**) The same TiO image as (**b**) but superimposed with arrows illustrating horizontal plasma flows tracked with DAVE (differential affine velocity estimator) and averaged for the time period 19:00:04–19:23:49 UT. The length of arrows denotes the flow magnitude, and for clarity, arrows in positive and negative magnetic fields are coded in red and yellow, respectively. The two white hollow arrows indicate the overall converging and shearing motions of the opposite polarity sunspot regions. The full TiO image sequence is provided in Supplementary Movie 4.

Disentangling the topology of the SFB as observed with BBSO/NST in projection is crucial for understanding the nature and properties of the flux rope. However, vector magnetic fields of higher atmosphere (for example, chromosphere and corona) are not routinely measured with the present instrument. To shed light on the three-dimensional magnetic field structure, we thus construct a NLFFF model at the pre-event time and plot some selected representative field lines in Fig. 3. Obviously, the extrapolated field lines coloured blue bear a reasonable resemblance to the morphology of the SFB seen in H-alpha (see Fig. 1b), showing in general two branches of arcade loops (AB and CD) that are highly sheared when passing each other along the PIL (we note that there may not exist a clear separation between the AB and CD domains). Importantly, we also find that below the blue field lines, there lies a group of longer field lines (coloured red) that directly connect the two ends of the SFB from A to D. These field lines AD might correspond to the loops observed in He I 10,830 Å as well as H-alpha offbands at a lower height (see Fig. 2a–c), as also clearly demonstrated in Fig. 4a. A side view of the red field lines AD is presented in Fig. 3b. On the basis of the force-free field extrapolations, we can further derive the magnetic twist number of a field line as 
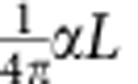
, where *α* is the force-free parameter and *L* is the field line length. It is obtained that the mean twist number of loops AB and CD is about 0.8 turns and that of AD is about 1.1 turns. Therefore, we speculate that the configuration of the SFB may represent a transition from twisted loops or flux ropes (for example, AD) to sheared arcades (for example, AB and CD). This may also be implied by the high-resolution BBSO/NST H-alpha images. We caution, however, that the NLFFF approach has limitations. These include that it does not take account of the plasma, and that it cannot be used to model the process of magnetic reconnection that involves non-force-free field.

### Confined motion

**Figure 5:**
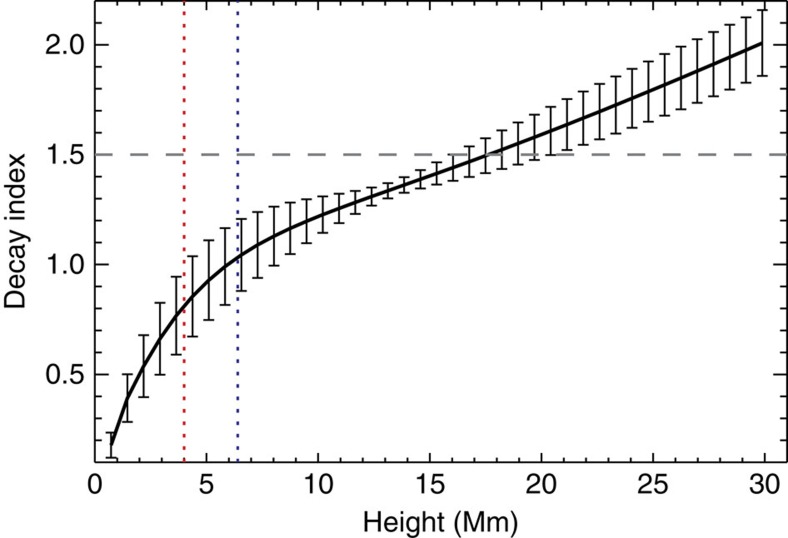
Decay profile of overlying field. We average the decay index at a certain height above the portion of the PIL beneath the flux rope (plotted as the white dotted line in Fig. 3), and plot the mean value versus height as the black line with the error bars representing 1 s.d. The red and blue dotted lines correspond to the maximum height of the red (flux rope) and blue (sheared arcades) field lines plotted in Fig. 3, respectively. The grey dashed line denotes the critical decay index value of 1.5 related to the torus instability condition.

It is worth mentioning that the aforementioned flux rope activity was not observed to develop into a coronal mass ejection. This suggests that the flux rope motion may be confined or failed. To shed light on this phenomenon, we calculate the decay index, defined by *n*=−dlog(*B*)/dlog(*h*), which can gauge the likelihood of eruption in the torus instability model. Here *B* is the strength of the horizontal component of the overlying potential field, *h* is the height above the surface and *n*>1.5–2.0 is the critical instability condition^24^. The calculation is performed above locations of the central portion of the PIL (the white dotted line in Fig. 3) underlying the flux rope. From the result plotted in Fig. 5, we can see that a flux rope would experience a decay index of the critical value at about 18 Mm, which is much higher than the maximum height (∼4 Mm) of the present flux rope of interest. This means that the magnetic field overlying the flux rope may have a strong strapping effect that prevents a full flux rope eruption. Nevertheless, the twisted flux rope may reconnect with the ambient field and reform as a less twisted field^25^,^26^.

## Discussion

**Figure 6:**
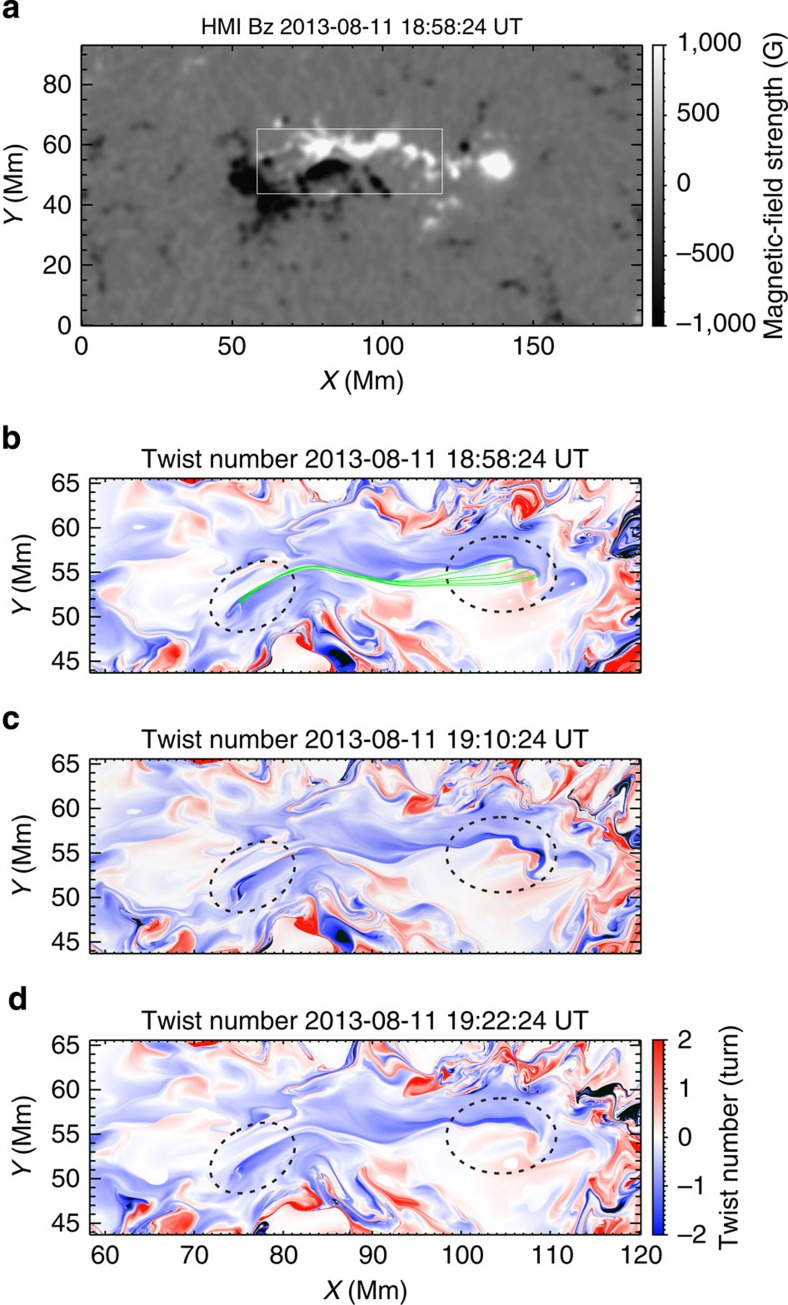
Evolution of magnetic twist. Maps of twist number are obtained by integrating the force-free parameter *α* along field lines divided by 4*π*. Only considered are field lines with both footpoints located within the field of view shown in (**a**). The white rectangle in **a** indicates the region in which the maps of twist number (**b**–**d**) are calculated. Green curves show exemplary twisted field lines in the map at 18:58:24 UT. Note a transient enhancement in the twist number at 19:10:24 UT at the footpoint regions of these field lines within the dotted ellipses.

On the basis of the BBSO/NST images and the NLFFF model result, we infer possible scenarios for this flux rope activity. The most likely explanation is that the twisted flux rope (modelled as loops AD underlying the SFB structure) may become unstable due to kink instability. Justifications for this view are discussed as follows. First, we show in Fig. 6 the maps with a larger field of view of twist number obtained by integrating the force-free parameter *α* along field lines. Note that a transient enhancement in the twist number at the footpoint regions (within the dotted ellipses) of the field lines similar to AD occurred at 19:10:24 UT, for a period before the onset of the event. Therefore, the sub-photospheric motion may play a significant role in enhancing the twist and destabilizing the flux rope. Second, the NLFFF model indicates the existence of such a flux rope, with a twist number comparable to that visualized in the high-resolution BBSO/NST observation. Third, it is found that on the surface, the opposite magnetic polarities of this active region exhibit an overall converging and shearing motions, which may be favourable for the gradual build-up of flux ropes^27^. These motions are obvious when observing the TiO time sequence images (see Fig. 4b and Supplementary Movie 4) and are clearly shown in the derived flow field (see Fig. 4c). Fourth, the interaction between this rising flux rope and the SFB can explain the brightenings of the flare ribbons with the standard two-ribbon flare model.

However, it is also possible that the magnetic reconnection between the sheared arcades contributes to the active flux rope. Specifically, a magnetic reconnection may occur between sheared arcades^17^,^27^ loosely denoted here as AB and CD. The main evidence is that the site of coronal magnetic reconnection, although not directly visible, may be manifested by a strong HXR-emitting source (the central contours in Fig. 1f) imaged by RHESSI around the flare peak. This source is located apparently between AB and CD, consistent with the speculated occurrence of reconnection. In the meantime, compact HXR emissions (left and right contours in Fig. 1f) are also produced near the two footpoints A and D, indicating enhanced precipitating electrons around the flare maximum. The weakening of the arcade structure before the flare, as shown in Fig. 1a,b, is also consistent with this view. Nevertheless, the sheared arcades still have a maximum height (∼6 Mm) well below the torus instability regime (see Fig. 5).

In conclusion, high-resolution images obtained by BBSO/NST make it possible to witness, for the first time, the detailed structure and evolution of a twisted flux rope in the low solar atmosphere. Observations with such a high resolution are invaluable in the studies of flux ropes, a magnetic structure important for energetic solar and interplanetary phenomena.

## Methods

### Observations and data processing

The essential observational evidence of the magnetic flux rope is provided by BBSO/NST. The telescope has gone through a few upgrades for its focal plane instrument and adaptive optics system. The latest upgrade of the high-order adaptive optics system uses 308 sub-apertures, which means that the telescope aperture is divided into 308 small telescopes so as to compensate and correct the atmospheric disturbances. Together with the speckle image reconstructions^28^, diffraction-limited imaging can thus be achieved. Here we use data (as shown in Fig. 1a–e, Fig. 2a,b, Fig. 4a and Supplementary Movies 1–3) from the Visible Imaging Spectrometer (VIS) at BBSO, which saw its first light with NST in the summer of 2013. This Fabry–Pérot filter-based system scans cross spectral lines in the range of 5,500–7,000 Å with millisecond steps. The H-alpha images have a spatial resolution of ∼0.068″ (50 km), and the cadence is 15 s for VIS to complete a scan at a 0.2 Å step from −1.0 to +1.0 Å around the H-alpha line centre. In addition, we also use He I 10830 Å images (as shown in Fig. 2c–f) taken by the InfraRed Imaging vector Magnetograph^29^,^30^ (IRIM) at BBSO and the broadband TiO (a proxy for the photosphere at 7,057 Å) images (as shown in Fig. 4b and Supplementary Movie 4). The cadence is 15 s and the spatial resolution is about 120 km for 10,830 Å and 65 km for the TiO data. While H-alpha off-bands and line-centre images cover a wide height range (∼1,500–3,000 km), the He I 10,830 Å line is formed around 2,000 km above the photosphere. The time sequence of TiO images can help to examine the photospheric flows (as shown in Fig. 4c), which are traced with the differential affine velocity estimator^31^, with the window size set to 19 pixels for feature tracking. Moreover, context information on the magnetic field structure of the solar photosphere (as shown in Fig. 1f) is provided by the vector magnetograms taken by the HMI instrument on the Solar Dynamic Observatory.

Signatures of magnetic reconnection can be revealed using the HXR emission due to the accelerated electrons, which was recorded for this flare event by RHESSI. The image of HXR sources, presented as contours in Fig. 1f, is reconstructed with the PIXON algorithm^32^ using detectors 1 and 3–8, and is integrated from 19:29:45 to 19:30:45 UT in the 25–50 keV energy band.

### Magnetic field modelling

The magnetic field model, as presented in Figs 3 and 4a, is constructed using the NLFFF extrapolation technique^21^. To obtain a chromospheric-like boundary data satisfying the force-free condition, a pre-processing procedure^33^ is first applied to the 2 × 2 rebinned Space-Weather HMI Active Region Patches^34^ data. Then the three-dimensional magnetic field is extrapolated using a ‘weighted optimization' method^35^,^36^. The extrapolation calculation is performed within a box of 584 × 248 × 256 uniform grid points, which corresponds to about 426 × 181 × 187 Mm^3^.

## Author contributions

H.W. developed the ideas for this study, coordinated the efforts of data analysis, wrote the first draft and led the discussion. W.C. developed instruments and led the observation at BBSO. C.L. contributed to the ideas, carried out the main data analysis and made major revisions of the manuscript. Y.X. contributed to the RHESSI data analysis. R.L. studied the magnetic twist and contributed to the scientific ideas. Z.Z. participated in the observations. J.C. contributed to the physical interpretation of the results. H.J. contributed the instrumentation of the 10830 observation. All authors participated in discussions, read and commented on the manuscript.

## Additional information

**How to cite this article:** Wang, H. *et al.* Witnessing magnetic twist with high-resolution observation of the 1.6-m New Solar Telescope. *Nat. Commun.* 6:7008 doi: 10.1038/ncomms8008 (2015).

## Supplementary Material

Supplementary Movie 1 BBSO/NST VIS observation at the H-alpha line centre. This video shows an active flux rope that appears to peel off from an overall inverse S-shaped flux bundle in NOAA Active Region 11817 on 2013 August 11. The field of view is about 32 Mm by 21 Mm. The image cadence is about 15 s, and the duration of the video is about 41 minutes.

Supplementary Movie 2 BBSO/NST VIS observation at the H-alpha blue wing (- 0.6 Å). The single flux rope structure before the event is more prominently shown compared to the H-alpha centre-line images.

Supplementary Movie 3 BBSO/NST VIS observation at the H-alpha red wing (+ 0.6 Å). The single flux rope structure before the event is more prominently shown compared to the H-alpha centre-line images.

Supplementary Movie 4 BBSO/NST observation in TiO. This video shows the overall converging and shearing motions of the opposite magnetic polarities of this active region (also see the derived flow field in Fig. 4c). The TiO images are aligned with H-alpha images shown in Supplementary Movies 1-3.
